# Supplementary material for: Proteomic analysis reveals shared biological pathways linking acrolein to biomolecular changes in the acute phase of rat spinal cord injury
Source: bioRxiv. 2026 Mar 14:2026.03.11.711153. Preprint. [Version 1] doi: 10.64898/2026.03.11.711153 (PMC13061048; doi:10.64898/2026.03.11.711153)
Supplement: Supplement 2 [file NIHPP2026.03.11.711153v1-supplement-2.pdf]

bioRxiv preprint doi: https://doi.org/10.64898/2026.03.11.711153; this version posted March 14, 2026. The copyright holder for this preprint (which was not certified by peer review) is the author/funder, who has granted bioRxiv a license to display the preprint in perpetuity. It is made available under aCC-BY 4.0 International license.

SUPPLEMENTARY FIGURES

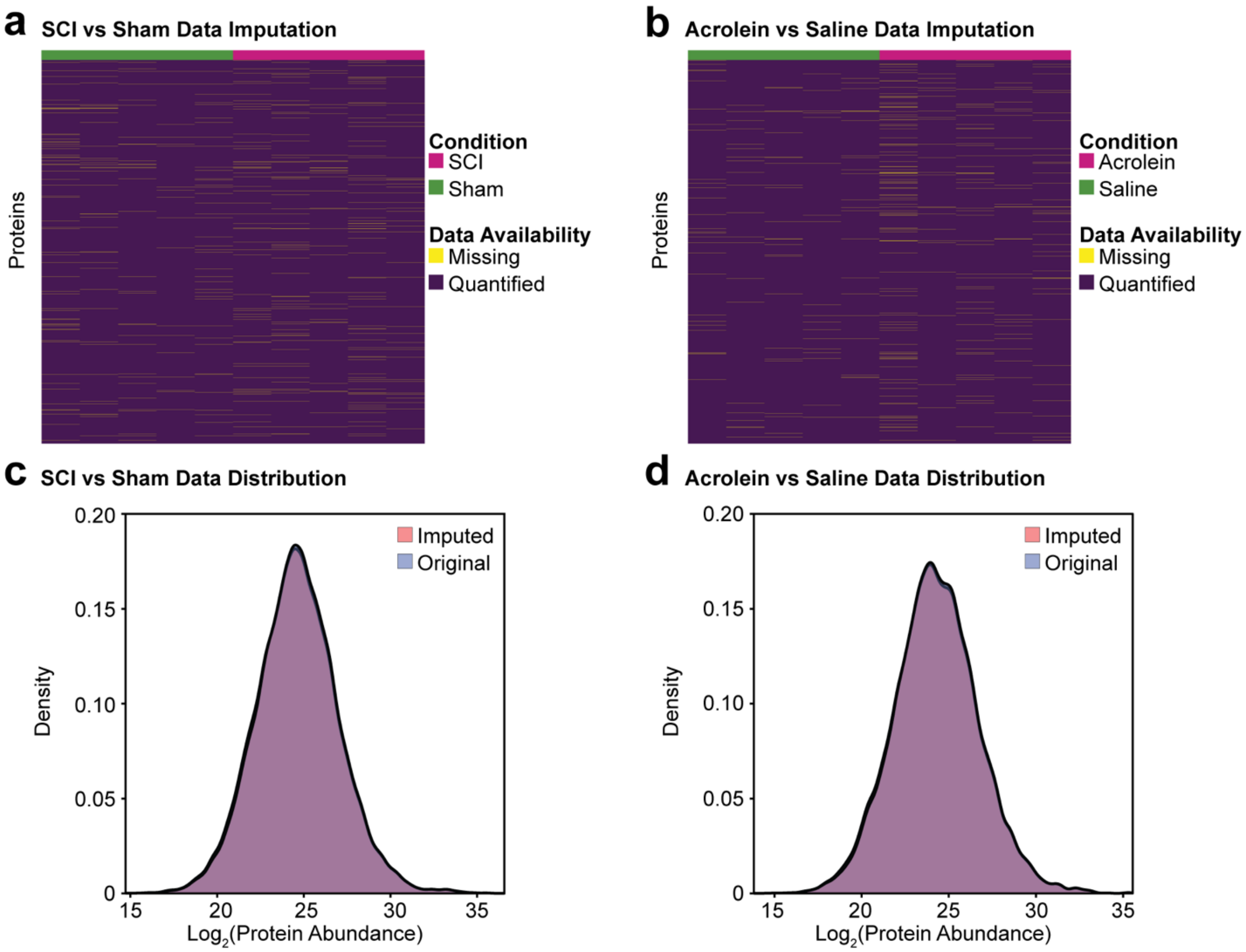

Supplementary Figure S1. Pre-processing of the proteomics data. (a) Data imputation of SCI and sham data. **(b)** Data imputation of the acrolein and saline injection data. **(c)** Data distribution pre- and post-imputation for SCI. **(d)** Data distribution pre- and post-imputation for acrolein.
